# Supplementary material for: IDseq—An open source cloud-based pipeline and analysis service for metagenomic pathogen detection and monitoring
Source: Gigascience. 2020 Oct 15;9(10):giaa111. doi: 10.1093/gigascience/giaa111 (PMC7566497; doi:10.1093/gigascience/giaa111)
Supplement: giaa111_Supplemental_Files [file giaa111_supplemental_files.zip › FigureS1.pdf]

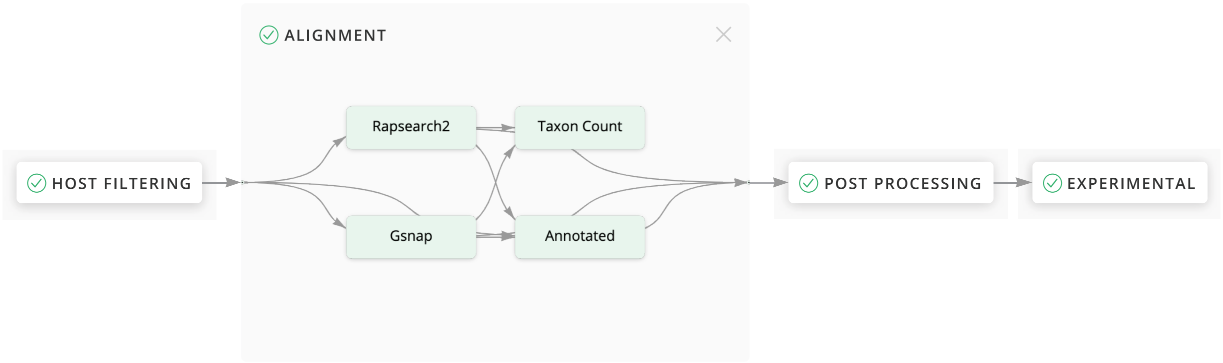
HOST FILTERING
ALIGNMENT
Rapsearch2
Taxon Count
Gsnap
Annotated
POST PROCESSING
EXPERIMENTAL
