# Supplementary material for: IDseq—An open source cloud-based pipeline and analysis service for metagenomic pathogen detection and monitoring
Source: Gigascience. 2020 Oct 15;9(10):giaa111. doi: 10.1093/gigascience/giaa111 (PMC7566497; doi:10.1093/gigascience/giaa111)
Supplement: giaa111_Supplemental_Files [file giaa111_supplemental_files.zip › giaa111_Supplemental_File.docx]

**SUPPLEMENTAL TEXT**

**Supplemental Methods**

*Internal Benchmark Datasets and Analysis*

Many closely related bacterial species have different relative pathogenicity in humans or other host organisms [1]. The unambiguous benchmark datasets, evaluated in the main text, do not emphasize performance at distinguishing common clinically relevant pathogens and closely related bacterial species. Here, we provide the results of an analysis of two simulated datasets containing common clinical microbes. For each dataset, we evaluated the performance of IDseq (NT and NR), as compared to Kraken2 [2], using per-species recall.

*Internal Benchmarks - Common Clinical Microbes (CCM) Benchmark Dataset*

Several microbes commonly identified in samples from humans and vector samples were identified in collaboration with researchers and incorporated into the CCM benchmark dataset. The selected species represent a range of bacterial, viral, fungal, and parasitic pathogens. The idseq-bench simulation tool was used to pull reference genomes from 35 specified accessions across 13 species (*Species name*, taxonomic ID; *Klebsiella pneumoniae*, 573; *Aspergillus fumigatus* Af293, 746128; *Plasmodium falciparum*, 5833; Rubella virus, 11041; Human immunodeficiency virus 1, 11676; Rhinovirus C, 463676; Chikungunya virus, 37124; *Staphylococcus aureus*, 1280; *Balamuthia mandrillaris*, 66527; *Elizabethkingia anophelis*, 1117645; *Neisseria meningitidis*, 487; Torque teno midi virus, 2065052; Hubei mosquito virus 2, 1922926 ) and simulate reads using the InSilicoSeq [3] sequence simulator with uniform coverage and sequencing error models derived from HiSeq data. The simulated sample is available on IDseq (**Table S3**, <https://idseq.net/ak8qu>). This simulated dataset is utilized by the IDseq team to evaluate the consistency of IDseq results over time and ensure the reliability of the pipeline results.

For IDseq NT and NR results, the idseq-bench score function was used to evaluate the per-read, species-level recall across each ground truth organism. Then, to characterize false positives, the total number of species identified was determined, along with the percentage of reads mapping to incorrect taxa. For Kraken2, the read-level recall was determined at the species level and reads mapping to higher taxonomic levels were not considered as positive matches for the species-level recall. Again, the total number of unique species was determined and the proportion of reads mapping incorrectly at the species level was determined.

*Internal Benchmarks - Closely Related Bacteria (CRB) Benchmark Dataset*

Many bacterial pathogens are closely related, therefore resulting in possible genomic overlap and ambiguity in aligning short read sequences that may map to multiple species. However, distinction of pathogens at the species-level has implications for treatment and outcomes. 10 bacterial species from 6 genera within the Enterobacteriaciae family were identified and the idseq-bench simulation tool was used to pull reference genomes (*Species name*, taxonomic ID; *Salmonella enterica*, 28901; *Citrobacter koseri*, 545; *Citrobacter freundii*, 546; *Klebsiella aerogenes*, 548; *Enterobacter cloacae*, 550; *Escherichia coli*, 562; *Klebsiella oxytoca*, 571; *Klebsiella pneumoniae*, 573; *Shigella boydii*, 621; *Shigella flexneri*, 623) and simulate reads using the InSilicoSeq [3] sequence simulator with uniform coverage and sequencing error models derived from HiSeq data. The simulated sample is available on IDseq (**Table S3**, <https://idseq.net/71wns>). Analysis of the CRB benchmark dataset replicated that which was done for the CCM dataset (above).

**Supplemental Results**

*External Benchmarks - NYCSM Outlier Dataset and IDseq NT*

The NYCSM dataset had the lowest AUPR of all datasets tested by IDseq NT. Overall, we note that due to the relative comparable performance of tools on these particular datasets, distinctions in AUPR may be the result of just one or two missed taxa. In this case, IDseq NT failed to identify *Enterobacter asburiae* (taxID = 61645) and identified only a small number (< 10) of reads aligning to *Pseudoalteromonas haloplanktis* (taxID = 228). Meanwhile, IDseq NR identified successfully recovered reads from *E. asburiae* and produced a comparably low number of reads mapping to *P. haloplanktis*. IDseq NT did identify many reads mapping to *Enterobacter soli*, which has been noted to have high genomic similarity to *E. asburiae* and *E. aerogenes* (p-distance: 1.06 and 1.19%, respectively [4]), both of which were included in the simulated dataset. This highlights the challenge with disambiguating short reads from taxa with a high degree of genomic similarity. We emphasize the practical importance of orthogonal validation of hits via assays (ie PCR) targeting unique regions of the genome. However, it is possible that the expansion of reference databases has improved specificity beyond the original dataset simulation. The particular GenBank accession to which all *E. soli* reads map (CP003026.1 Enterobacter soli strain LF7a, complete genome), was added to the database in April 2019, while reads were simulated in 2016 [5]. The same trend is observed for *P. haloplanktis*. IDseq NT and NR both identified significantly more reads mapping to *P. arctica*, a member of the *P. haloplanktis*-like group [6]. The associated GenBank accession to which the majority of reads map (CP011025.1 - Pseudoalteromonas arctica A 37-1-2 chromosome I, complete sequence) was added in September of 2017. The IDseq coverage visualization makes interrogation of microbial hits simple by linking to the NCBI taxonomy database and GenBank accessions.

*Viral Divergence in Recent Human Disease*

The ability to detect divergent viruses is a function of genomic similarity to other organisms in the database as well as genomic coverage, which influences assembled contig lengths. Many recent, emerging, diseases affecting humans do have some sequence similarity to organisms in the NCBI databases. For example, since the emergence of enterovirus EV-D68, numerous outbreaks caused by divergent sub-clades have been reported with nt similarity to other strains ~96% [7]. Similarly, recent outbreaks of Dengue virus have been reported to be the result of introduction of novel DENV lineages, which are defined based on nucleotide divergence of 6-8% within each DENV serotype[8,9]. The set of nine known West Nile Virus lineages (which genomic analysis indicated diverged in the early 17th century) have an average pairwise percent identity of 77.6% (nucleotide) and 90.1% (amino acid) [10]. These are well within the range for detection by IDseq. Meanwhile, Zika virus, the viral species which caused the recent 2015 epidemic, was discovered in 1940 and shares, on average, 55.6% amino acid sequence identity with dengue virus and 57.0% with West Nile virus [11]. Had the sequence of the first Zika isolate not been in the database, IDseq would not have been able to flag the presence of this virus and researchers would be required to evaluate assembled, unmapped, contigs offline [12].

*Internal Benchmark Datasets Results*

Two additional datasets were simulated to evaluate IDseq’s performance for detection of common clinical microbes (CCM) as well as for disambiguating closely related bacterial species (CRB) (Methods, **Table S3**). The CCM dataset contains reads simulated from 13 total species, including six viral, four bacterial, and three eukaryotic pathogens. The CRB dataset contains reads simulated from 10 species of bacteria, all from the Enterobacteriaceae family. Given that IDseq’s pipeline returns a species-level assignment for each mapped read and the web interface presents results at the species level, we evaluate each of these benchmarks only at the species level. The Kraken2 algorithm assigns reads with ambiguous mappings to higher taxonomic levels. Therefore, the results shown include only the species-specific alignment results.

For the CCM dataset, IDseq filtered out 15.3% of reads during the host and QC filtering pipeline steps. Of the remaining 99,320 non-host reads, IDseq NT showed the greatest per-species recall across the 13 species included (1.0, IQR = 0.99 – 1.0). IDseq NT per-species recall was significantly higher than that of both IDseq NR (0.87, IQR = 0.85 – 1.0, p = 0.02) and Kraken2 (0.89, IQR = 0.64 – 1.0, p = 0.005 Wilcoxon rank sum) (**Figure S3A**). All tools successfully identified the presence of all 13 microbial species. In addition to these species, IDseq NT, NR, and Kraken2 identified 63, 303, and 230 false positive species, respectively. Since the IDseq pipeline returns a species-level assignment for all mapped reads, even in cases where the species may align equally to two different species, it had a notably greater portion of the total (post-qc) reads mapping across those false positive organisms (3.0 % by nt, 10.0 % by NR) than Kraken2, which had only 0.56 % of reads mapping to the false positive species. The number of reads per false positive organism was low (mean = 66 reads by nt, mean = 41 reads by nr), highlighting the utility of filtering in the IDseq web application. Kraken2 avoids larger percentages of reads being associated with false-positive species calls by calling a significant portion of ambiguously mapped reads at higher levels of the taxonomic tree.

Similar trends were observed for the CRB dataset. IDseq filtered out 15.6% of reads during the host and QC filtering pipeline steps, leaving 84,410 non-host reads for down-stream analysis. Notably, IDseq nt demonstrates the highest per-species recall (0.68, IQR = 0.43 – 0.80), significantly different than NR (0.46, IQR = 0.15 – 0.61, p = 0.005, Wilcoxon rank sum) and Kraken2 (0.18, IQR = 0.11 – 0.35, p = 0.005, Wilcoxon rank sum) (**Figure S3B**). All tools successfully identified the presence of all 13 microbial species. In addition to these species, IDseq NT, NR, and Kraken2 identified 83, 371, and 167 false positive species, respectively. Again, IDseq NT and NR had greater proportions of total reads mapping to these false-positive species (31.7% and 49.7% for NT and NR, respectively) as compared to Kraken2, with only 0.6 % of reads mapping to false-positive species and the majority of ambiguous reads mapping at higher levels of classification (70.9%).

The impact of ambiguous reads is exaggerated in cases where we simulate reads from multiple closely related species with known genomic similarity. In many cases of an infection, with a single dominant organism, the importance of recall may outweigh the identification of lower-level false-positive species. Additionally, these simulations sample from across the genome. However, we know that ribosomal sequences can be used for typing of bacteria and studies have previously shown improved sensitivity with RNA-seq, where ribosomal RNA comprises the greatest portion of sequenced nucleic acid [13,14]. Thus, IDseq results must be interpreted by the researcher with respect to the sample type and sequencing prep.

**Supplemental Figures**

**Figure S1:** The IDseq pipeline visualization indicates each step in the underlying pipeline and includes a description of the raw command parameters as well as the ability to download intermediate files for offline analysis.

**Figure S2:** Performance metrics evaluated across 20 mNGS taxonomic identification tools, **A)** Area under the precision recall curve (AUPR), ranges from 0 to 1 (best), **B)** Precision, ranges from 0 to 1 (best). **C)** Recall, ranges from 0 to 1 (best) **D)** F1-score, the harmonic mean of precision and recall values. **E)** L2 distance, ranges from 0 (best) to 1.

**Figure S3:** Per-species recall values for two internal benchmark datasets, **A)** The common clinical microbes (CCM) dataset and **B)** the closely related bacteria (CRB) dataset.

**Supplemental Tables**

**Table S1:** Github repositories containing open-source code for IDseq pipeline, web application, and benchmarking resources.

| **Tool** | **Code Location** |
| --- | --- |
| IDseq Pipeline | <https://github.com/chanzuckerberg/idseq-dag> |
| IDseq Web Application | <https://github.com/chanzuckerberg/idseq-web> |
| IDseq Benchmarking Tool | <https://github.com/chanzuckerberg/idseq-bench> |

**Table S2:** External benchmark datasets and their corresponding IDseq links.

| **Sample Name** | **IDseq Link** |
| --- | --- |
| UnAmbiguouslyMapped_ds.7 | <https://idseq.net/itivl> |
| UnAmbiguouslyMapped_ds.buccal | <https://idseq.net/zczoz> |
| UnAmbiguouslyMapped_ds.cityparks | <https://idseq.net/rp9jd> |
| UnAmbiguouslyMapped_ds.gut | <https://idseq.net/358id> |
| UnAmbiguouslyMapped_ds.hous1 | <https://idseq.net/rjmux> |
| UnAmbiguouslyMapped_ds.hous2 | <https://idseq.net/gzznk> |
| UnAmbiguouslyMapped_ds.nycsm | <https://idseq.net/jagjg> |
| UnAmbiguouslyMapped_ds.soil | <https://idseq.net/pbs4k> |
| atcc_even | <https://idseq.net/bkwxo> |
| atcc_staggered | <https://idseq.net/profp> |

**Table S3:** Internal benchmark datasets and their corresponding IDseq links.

| **Sample Name** | **Project Name** | **Benchmark Description** | **IDseq Link** |
| --- | --- | --- | --- |
| HRC_100 | HRhinoC Simulation | Divergent Rhinovirus C, 100% identity to reference | <https://idseq.net/lx7cf> |
| HRC_099 | HRhinoC Simulation | Divergent Rhinovirus C, 99% identity to reference | <https://idseq.net/1a3we> |
| HRC_095 | HRhinoC Simulation | Divergent Rhinovirus C, 95% identity to reference | <https://idseq.net/7a0he> |
| HRC_090 | HRhinoC Simulation | Divergent Rhinovirus C, 90% identity to reference | <https://idseq.net/8gmj7> |
| HRC_085 | HRhinoC Simulation | Divergent Rhinovirus C, 85% identity to reference | <https://idseq.net/q4wks> |
| HRC_080 | HRhinoC Simulation | Divergent Rhinovirus C, 80% identity to reference | <https://idseq.net/vf8hm> |
| HRC_075 | HRhinoC Simulation | Divergent Rhinovirus C, 75% identity to reference | <https://idseq.net/qje7h> |
| HRC_070 | HRhinoC Simulation | Divergent Rhinovirus C, 70% identity to reference | <https://idseq.net/s299t> |
| HRC_065 | HRhinoC Simulation | Divergent Rhinovirus C, 65% identity to reference | <https://idseq.net/n6ndl> |
| HRC_060 | HRhinoC Simulation | Divergent Rhinovirus C, 60% identity to reference | <https://idseq.net/46kfh> |
| HRC_055 | HRhinoC Simulation | Divergent Rhinovirus C, 55% identity to reference | <https://idseq.net/hggw6> |
| HRC_050 | HRhinoC Simulation | Divergent Rhinovirus C, 50% identity to reference | <https://idseq.net/a8z8a> |
| HRC_045 | HRhinoC Simulation | Divergent Rhinovirus C, 45% identity to reference | <https://idseq.net/64ita> |
| HRC_040 | HRhinoC Simulation | Divergent Rhinovirus C, 40% identity to reference | <https://idseq.net/guat0> |
| HRC_035 | HRhinoC Simulation | Divergent Rhinovirus C, 35% identity to reference | <https://idseq.net/shygl> |
| HRC_030 | HRhinoC Simulation | Divergent Rhinovirus C, 30% identity to reference | <https://idseq.net/hdc6r> |
| HRC_025 | HRhinoC Simulation | Divergent Rhinovirus C, 25% identity to reference | <https://idseq.net/v84fc> |
| CCM Dataset | Benchmark v1 | Common clinical microbes, simulated with idseq-bench | <https://idseq.net/ak8qu> |
| CRB Dataset | Benchmark v2 | Closely related bacterial species, simulated with idseq-bench | <https://idseq.net/71wns> |

**Supplemental References**

1. Guentzel MN. Escherichia, Klebsiella, Enterobacter, Serratia, Citrobacter,and Proteus. *Med. Microbiol*. 1996.

2. Wood DE, Lu J, Langmead B. Improved metagenomic analysis with Kraken 2. *Genome Biol*. 2019;20:257.

3. Gourlé H, Karlsson-Lindsjö O, Hayer J, Bongcam-Rudloff E. Simulating Illumina metagenomic data with InSilicoSeq. [cited 2020 Feb 26]; Available from: https://github.com/HadrienG/InSilicoSeq

4. Manter DK, Hunter WJ, Vivanco JM. Enterobacter soli sp. nov.: A lignin-degrading γ-Proteobacteria isolated from soil. *Curr Microbiol*. 2011;62:1044–9.

5. Ounit R, Lonardi S. Higher classification sensitivity of short metagenomic reads with CLARK-S. *Bioinformatics*. 2016;32:3823–5.

6. Bosi E, Fondi M, Orlandini V, Perrin E, Maida I, de Pascale D, et al. The pangenome of (Antarctic) Pseudoalteromonas bacteria: Evolutionary and functional insights. *BMC Genomics*. 2017;18:93.

7. Pellegrinelli L, Giardina F, Lunghi G, Renteria SCU, Greco L, Fratini A, et al. Emergence of divergent enterovirus (EV) D68 sub-clade D1 strains, northern Italy, September to October 2018. *Eurosurveillance*. 2019;24.

8. Ahamed SF, Rosario V, Britto C, Dias M, Nayak K, Chandele A, et al. Emergence of new genotypes and lineages of dengue viruses during the 2012–15 epidemics in southern India. *Int J Infect Dis*. 2019;84:S34–43.

9. Jesus JG de, Dutra KR, Salles FC da S, Claro IM, Terzian AC, Candido D da S, et al. Early identification of dengue virus lineage replacement in Brazil using portable genomic surveillance. *bioRxiv*. 2019;716159.

10. Fall G, Di Paola N, Faye M, Dia M, Freire CC de M, Loucoubar C, et al. Biological and phylogenetic characteristics of West African lineages of West Nile virus. *PLoS Negl Trop Dis*. 2017;11:e0006078.

11. Chang HH, Huber RG, Bond PJ, Grad YH, Camerini D, Maurer-Stroh S, et al. Systematic analysis of protein identity between Zika virus and other arthropod-borne viruses. World Health Organization; 2017;95:517–25.

12. Batson J, Dudas G, Haas-Stapleton E, Kistler AL, Li LM, Logan P, et al. Single mosquito metatranscriptomics recovers mosquito species, blood meal sources, and microbial cargo, including viral dark matter. *bioRxiv*. 2020;2020.02.10.942854.

13. Ribosomal RNA - an overview | ScienceDirect Topics [Internet]. [cited 2020 Feb 26]. Available from: https://www.sciencedirect.com/topics/neuroscience/ribosomal-rna

14. Cottier F, Srinivasan KG, Yurieva M, Liao W, Poidinger M, Zolezzi F, et al. Advantages of meta-total RNA sequencing (MeTRS) over shotgun metagenomics and amplicon-based sequencing in the profiling of complex microbial communities. *Biofilms Microbiomes*. 2018;4:1–7.
