# Supplementary figures and images for: IDseq—An open source cloud-based pipeline and analysis service for metagenomic pathogen detection and monitoring
Source: Gigascience. 2020 Oct 15;9(10):giaa111. doi: 10.1093/gigascience/giaa111 (PMC7566497; doi:10.1093/gigascience/giaa111)

(AUPR) Area Under the  
Precision Recall Curve

Precision

Recall

F1-Score

L2 Distance

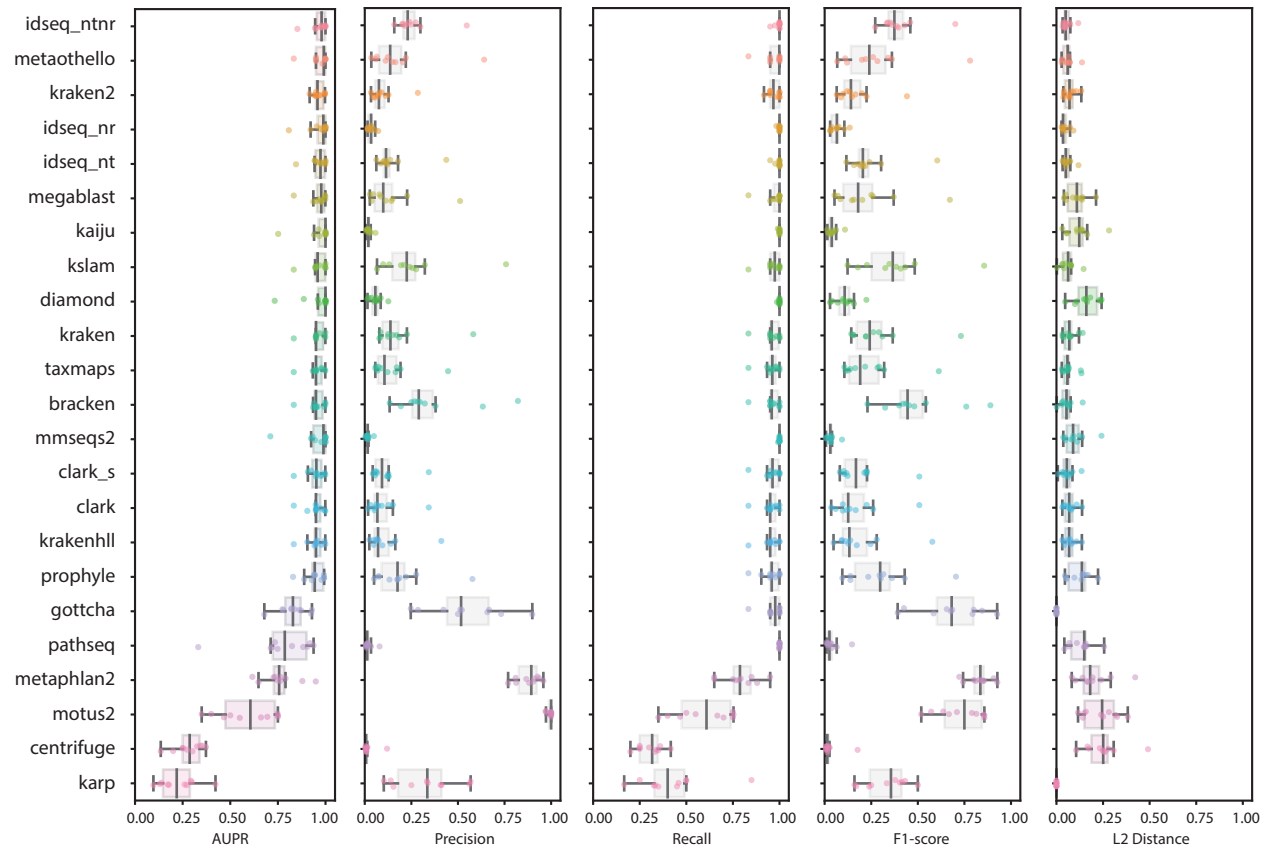

Supplement: giaa111_Supplemental_Files [file giaa111_supplemental_files.zip › FigureS2.pdf]

**A** CCM Dataset

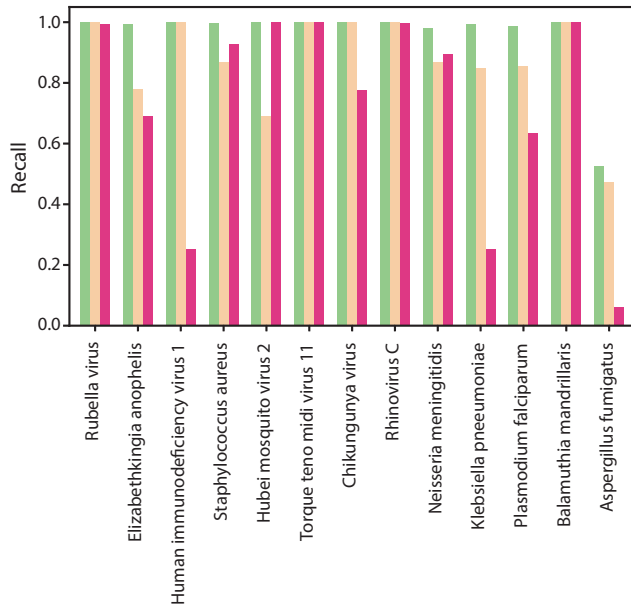

**B** CRB Dataset

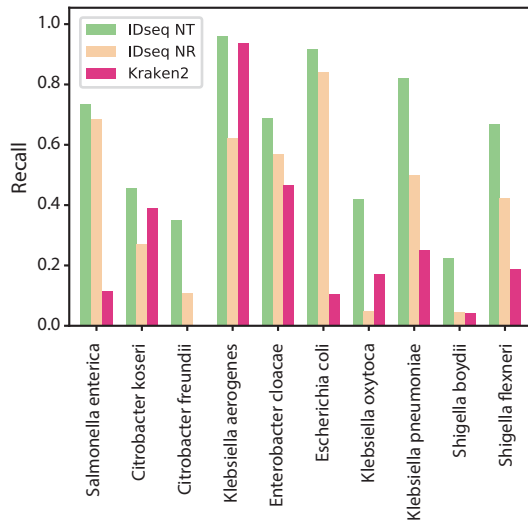

Supplement: giaa111_Supplemental_Files [file giaa111_supplemental_files.zip › FigureS3.pdf]
