# Supplementary material for: IDseq—An open source cloud-based pipeline and analysis service for metagenomic pathogen detection and monitoring
Source: Gigascience. 2020 Oct 15;9(10):giaa111. doi: 10.1093/gigascience/giaa111 (PMC7566497; doi:10.1093/gigascience/giaa111)
Supplement: giaa111_Response_to_Reviewer_Comments_Original_Submission [file giaa111_response_to_reviewer_comments_original_submission.pdf]

Author's Response To Reviewer Comments

Close

Please find responses to each of the reviewer comments in-line below.

Reviewer #1:
The authors submitted a manuscript entitled: "IDseq - An Open Source Cloud-based Pipeline and Analysis Service for Metagenomic Pathogen Detection and Monitoring". The manuscript is well written and very clearly structured. The theory behind the tool is scientifically sound and benchmarking has been performed but I had wished for testing more than one SARS-Cov2 samples given the current pandemic (see below).
Overall, I recommend the manuscript to be accepted with minor revision.
Unfortunately, I can only form an opinion about the paper and not the tool at this moment. I would have liked to test the tool itself but ran into immediate problems trying to upload a large data file using the command line. First errors were solved quickly but when another error occurred the response from the support was unfortunately slow and advice of how to fix this error only came in this morning. As the deadline for the revision is today, I did not have enough time to test the tool.

Minor revision:

Comment #1: Please comment how you ensure that no human data can be exploited and that you follow international laws, including GDPR.

Response:
IDseq ensures that human data is removed from samples processed in the IDseq portal through three independent host filtering steps – the first is rapid host removal via STAR, then a second round of removal of host sequence using Bowtie2 configured for additional sensitivity, and finally a universal removal of human sequences (regardless of host organism) using Gsnap, which features BLAST-level sensitivity. For humans in particular, we use a combined host database comprised of Hg38 and Pan troglodytes, which can assist with removing human sequences not explicitly in Hg38. This is detailed in the manuscript on page 6 as such:

"IDseq performs a priori subtraction of host sequences via STAR (Spliced Transcripts Alignment to a Reference) alignment of raw reads to a host-specific database [26].”

"Regardless of the host genome, the data is scoured to remove all remaining human sequences using Bowtie2 against the HG38 reference database [31] and gmap-gsnap against a more stringent database including sequences combining both HG38 and Chimpanzees (Pan troglodytes) [32]. This step is especially important in the case of vector research, where blood meals may contain human sequences."

Furthermore, the IDseq terms of service (https://idseq.net/terms) outline the methods used to compliance with international laws regarding the use of human data, including GDPR.

Comment #2: Is this platform only useful for Illumina sequencing reads? Is it compatible with other platforms and platform-specific sequencing errors, e.g. MGI sequencing?

Response:
The IDseq platform currently only supports short-read sequencing data. We hope to expand to other platforms in the future. That said, on page 5, we specify:
“The IDseq pipeline ingests raw, short-read sequencing data (either RNA- or DNA-seq from any sample type), which can be uploaded from local sources via the web interface or the command line interface (CLI) or directly from Illumina’s BaseSpace platform.”

To provide additional clarity, we have updated the pipeline description on page 6:

“The first phase of the pipeline begins with validation of input files (single- or paired-end .fastq or .fasta files from short-read sequencing libraries).”

While other sequencing technologies are up-and-coming, the current majority of mNGS experiments are done using Illumina short-read sequencing data. As other sequencing technologies gain traction in the field, we will evaluate the ability to incorporate analysis of these data.

Comment #3: On page 13 you state: "In the context of pathogen-identification, it has been observed that infecting agents may comprise the majority of sequencing reads in certain circumstances." However, when it comes to viral infections in the respiratory tract, including, but not limited to CoV2, this is not the case. Please make a stronger statement of that and how IDseq works in these cases.

Response:
The reviewer makes a great point about respiratory viruses, which may be present at low abundance. We have amended the statement as follows:
“But use of the full NCBI database may result in false-positive alignments to related taxa at low abundance which can reduce precision (Figure 3C). This is especially true for bacterial taxa, with homology in the 16s rRNA regions. In the context of pathogen-identification, it has been observed that infecting agents mayoften comprise the majority of sequencing reads in certain circumstances [27]. For such data sets, the reduced precision for abundance estimation at low levels is less impactful. One case in which this may not be true is in the case of viral respiratory infection, whereby a small number of sequencing reads may be indicative of infection. In this case, targeted analysis using IDseq to filter for only viral reads will improve sensitivity. Meanwhile, researchers interested in evaluating highly complex microbiome composition at the species- and strain-level may need to bring in other tools to supplement their analyses [40–42] or rely on genus-level estimates provided by IDseq.”

Comment #4: Page 19: It is desirable to include more patient samples with Covid-19, with varying viral loads. Not many viral reads were found (which is in conflict with one of your statements above). So it is important to know whether this patient had very low or very high viral load. Where is the detection limit for IDseq, e.g. related to Ct values in qPCR commonly used in Covid-19 diagnostics, and what may be the false negative rate for e.g. SARS-Cov2 patients?

Response:
We appreciate the reviewers’ interest in more clarity on the limit of detection for SARS-CoV2 in patient samples. The requested information regarding the limit of detection of SARS-CoV-2 through the analysis of a greater number of COVID-positive samples is the subject of a much larger study of COVID-19 patients that is. ongoing. Thus, it is out of scope for this manuscript which serves to highlight the work of a single group in Cambodia and focuses on benchmarking the ability for the pipeline to identify novel organisms prior to their inclusion in the NCBI reference databases.

For this particular case, we have added further detail to the manuscript as follows:

“On January 30, 2020, a team of researchers from the CNM-NIAID (National Center for Parasitology, Entomology, and Malaria Control - National Institute of Allergy and Infectious Disease) collaboration in Cambodia obtained a nasopharyngeal swab sample from a patient with PCR-confirmed SARS-CoV-2 infection (Ct = 24).”

Other manuscripts have rigorously evaluated the limit of detection using IDseq and External RNA Controls Consortium (ERCC) spike-ins, which can be used to evaluate the total sample input mass and the limit of detection (Zinter et al. Microbiome 2019). The SARS-CoV-2 sample discussed in this manuscript was processed using ERCCs. In this sample, ERCC-00017 was the ERCC control detected (2 reads) with the lowest concentration (0.1144 attamoles/uL). It has been shown that the input mass is proportional the number of sequencing reads. This suggests that the lower limit of detection for this sample was approximately 0.06 attamoles/uL.

Comment #5: Page 24: Could you please explain further how the z-score is calculated if the taxonomic ID is in both sample and control but at different abundances?

Response:

We appreciate the complexities of interpreting taxons present in both samples and controls and the request for clarity around the z-score computation. We have added the additional details regarding the calculation and interpretation of the z-score in these cases.

"The z-score field of the IDseq sample report is calculated as the z-score for each taxonomic ID based on its prevalence in the selected background model. Specifically, the z-score for a taxon in sample A is computed as:
z= (x-μ)/σ= ((rpm of taxon in sample)-mean(rpm of taxon in background model samples))/(std_dev(rpm of taxon in background model samples)).
Thus, taxons present at higher abundance in the sample than the controls, it will have a z-score > 1. If a particular taxonomic ID is not found in the set of control samples, then the z-score will be set to 100. If the taxonomic ID is not found in the sample, the z-score will be set to -100."

Comment #6: Page 5 (Supplemental text): "Since the IDseq pipeline returns a species-level assignment for all mapped reads, even in cases where the species may align equally to two different species, it had a notably greater portion of the total (post-qc) reads mapping across those false positive organisms (3.0 % by nt, 10.0 % by NR) than Kraken2, which had only 0.56 % of reads mapping to the false positive species. Kraken2 avoids larger percentages of reads being associated with false-positive species calls by calling a significant portion of ambiguously mapped reads at higher levels of the taxonomic tree… Again, IDseq NT and NR had greater proportions of total reads mapping to these false-positive species (31.7% and 49.7% for NT and NR, respectively) as compared to Kraken2, with only 0.6 % of reads mapping to false-positive species and the majority of ambiguous reads mapping at higher levels of classification (70.9%)." I am concerned about these high false positive rates. Is there information in IDseq output to extract information on how many reads were ambiguous and to which species? This could be very important information for the user and then could be followed up with species-specific PCR or other tests.

Response:
We appreciate the reviewer's concern for false positives. As demonstrated through the benchmarking analyses, IDseq prioritizes sensitivity at the cost of reduced specificity. The metrics show the total percentage of reads mapping to false positive taxa. However, the majority of these taxa have few total reads. These challenges associated with low-abundance false positives due to taxonomic ambiguity in homologous regions can be addressed through the filtering abilities enabled in the web application (page 9).

"For all views of the data, a wide range of user-selectable compound query and filtering tools are made available, enabling facile investigation of the data."

We have clarified this in the text by adding further details regarding the distribution of these reads across taxa:

"Since the IDseq pipeline returns a species-level assignment for all mapped reads, even in cases where the species may align equally to two different species, it had a notably greater portion of the total (post-qc) reads mapping across those false positive organisms (3.0 % by nt, 10.0 % by nr) than Kraken2, which had only 0.56 % of reads mapping to the false positive species. The number of reads per false positive organism was low (mean = 66 reads by nt, mean = 41 reads by nr), highlighting the utility of filtering in the IDseq web application. Kraken2 avoids larger percentages of reads being associated with false-positive species calls by calling a significant portion of ambiguously mapped reads at higher levels of the taxonomic tree."

However, in cases where there are many closely related bacteria, genomic similarity is known to pose a challenge for most mNGS analysis tools. To address this, IDseq provides all intermediate files after host removal for download – this includes files with intermediate alignment results. At each alignment step (short-read alignment with GSNAP (nt) and rapsearch2 (nr), as well as BLAST alignment (nt and nr)), the intermediate alignments include all alignments for the read or contig. From this information, it is possible to evaluate whether a read or set of reads mapped uniquely to a particular taxon or mapped equally well across many taxons.

Reviewer #2:

Comment #1: Excellent work and need of the hour.

Response:
We appreciate this comment!

Reviewer #3:
Summary:
The goal of this study is to provide an open source cloud-based metagenomics pipeline and service for global pathogen detection and monitoring from raw next-generation sequencing (NGS) reads. Their platform is optimized for scalable Amazon Web Services (AWS) cloud deployment. The authors provided a portal, IDseq portal to get raw mNGS data as input and to generate the assignment of reads and contigs to taxonomic categories.
This paper mainly presents two key contributions:
1. Providing an open source portal for pathogen detection from raw next-generation sequencing (NGS).
2. Providing open source Github repos.

Comments:
The paper makes a good effort to introduce an open source platform for pathogen detection. However, the provided Github is not working on a cloud platform other than AWS. I chose two platform to test the Github code on powerful cloud severs, however, the coded seems not working. These are the samples of errors:

ERROR: test_many_samples (tests.test_samples_on_local_steps.TestSamplesOnLocalSteps)

tests/test_utils.py", line 84, in run_step_and_match_outputs
test_bundle, output_dir_s3)

idseq-dag/tests/idseq_step_setup.py", line 82, in get_test_step_object
command.make_dirs(result_dir_local)
mkdir(name, mode)
PermissionError: [Errno 13] Permission denied: '/mnt/idseq'

¬¬
We highly suggest the authors to fix these issue, and make their Github repo easy to work and install.

Response:

We appreciate the reviewer’s suggestion that we make it possible to run the IDseq pipeline separately from the web portal. We must first highlight that the IDseq portal is intended for an audience of researchers without access to significant computational experience and server-class hardware. Thus, the main entry-point to the tool is through the web portal. It is possible to get an account for evaluating the tool via http://idseq.net.

To the reviewer’s point, the web portal was initially designed to run on AWS architecture and was interwoven with dependencies which may have caused the errors indicated. We have noted the reviewer’s suggestion that we make it possible to run the IDseq pipeline on other infrastructures beyond AWS. To this point, we have undergone a significant project in updating the IDseq pipeline code to be more encapsulated and provide the ability to run on other infrastructures. Additionally, we have written a complete how-to, step-by-step document that should aid any user in executing the IDseq pipeline on a smaller database in a local environment. To enable this functionality, the original GitHub repositories have been ported to https://github.com/chanzuckerberg/idseq-workflows. As mentioned above, the default databases (NCBI NT and NR) are extremely large and it is not feasible to host such large files on GitHub. Regardless, this version of the pipeline can now be run on a smaller test database (composed of viral-only sequences) and local computational resources without the front-end portal by following the instructions found here: https://github.com/chanzuckerberg/idseq-workflows/wiki/Running-WDL-workflows-locally. The user may expand the database, of course, on their own. For the purposes of evaluating the software, the provided viral-only sequence database should be sufficient. Note that the pipeline version is running online today is v4.11. The manuscript still reflects results for version 3.13. The changes between these versions are documented here: https://github.com/chanzuckerberg/idseq-

dag#release-notes. While these differences may change the individual numbers, the overall performance characteristics of the pipeline remain the same. I do not expect these differences to significantly impact any of the conclusions drawn in this paper.

Close
