# Supplementary material for: IDseq—An open source cloud-based pipeline and analysis service for metagenomic pathogen detection and monitoring
Source: Gigascience. 2020 Oct 15;9(10):giaa111. doi: 10.1093/gigascience/giaa111 (PMC7566497; doi:10.1093/gigascience/giaa111)
Supplement: giaa111_Reviewer_1_Report_Original_Submission — Stefanie Prast-Nielsen, Ph.D. -- 5/8/2020 Reviewed [file giaa111_reviewer_1_report_original_submission.pdf]

Reviewer Report

Title: IDseq â€“ An Open Source Cloud-based Pipeline and Analysis Service for Metagenomic Pathogen Detection and Monitoring

Version: Original Submission **Date:** 5/8/2020

Reviewer name: Stefanie Prast-Nielsen, Ph.D.

Reviewer Comments to Author:

The authors submitted a manuscript entitled: "IDseq - An Open Source Cloud-based Pipeline and Analysis Service for Metagenomic Pathogen Detection and Monitoring". The manuscript is well written and very clearly structured. The theory behind the tool is scientifically sound and benchmarking has been performed but I had wished for testing more than one SARS-Cov2 samples given the current pandemic (see below).
Overall, I recommend the manuscript to be accepted with minor revision.
Unfortunately, I can only form an opinion about the paper and not the tool at this moment. I would have liked to test the tool itself but ran into immediate problems trying to upload a large data file using the command line. First errors were solved quickly but when another error occurred the response from the support was unfortunately slow and advice of how to fix this error only came in this morning. As the deadline for the revision is today, I did not have enough time to test the tool.
Minor revision:
Please comment how you ensure that no human data can be exploited and that you follow international laws, including GDPR.
Is this platform only useful for Illumina sequencing reads? Is it compatible with other platforms and platform-specific sequencing errors, e.g. MGI sequencing?
On page 13 you state: "In the context of pathogen-identification, it has been observed that infecting agents may comprise the majority of sequencing reads in certain circumstances." However, when it comes to viral infections in the respiratory trackt, including, but not limited to CoV2, this is not the case. Please make a stronger statement of that and how IDseq works in these cases.
Page 19: It is desirable to include more patient samples with Covid-19, with varying viral loads. Not many viral reads were found (which is in conflict with one of your statements above). So it is important to know whether this patient had very low or very high viral load. Where is the detection limit for IDseq, e.g. related to Ct values in qPCR commonly used in Covid-19 diagnostics, and what may be the false negative rate for e.g. SARS-Cov2 patients?
Page 24: Could you please explain further how the z-score is calculated if the taxonomic ID is in both sample and control but at different abundances?
Page 5 (Supplemental text): "Since the IDseq pipeline returns a species-level assignment for all mapped reads, even in cases where the species may align equally to two different species, it had a notably greater portion of the total (post-qc) reads mapping across those false positive organisms (3.0 % by nt, 10.0 % by NR) than Kraken2, which had only 0.56 % of reads mapping to the false positive species. Kraken2 avoids larger percentages of reads being associated with false-positive species calls by calling a

significant portion of ambiguously mapped reads at higher levels of the taxonomic tree… Again, IDseq NT and NR had greater proportions of total reads mapping to these false-positive species (31.7% and 49.7% for NT and NR, respectively) as compared to Kraken2, with only 0.6 % of reads mapping to false-positive species and the majority of ambiguous reads mapping at higher levels of classification (70.9%)." I am concerned about these high false positive rates. Is there information in IDseq output to extract information on how many reads were ambiguous and to which species? This could be very important information for the user and then could be followed up with species-specific PCR or other tests.

Level of Interest

Please indicate how interesting you found the manuscript: Choose an item.

Quality of Written English

Please indicate the quality of language in the manuscript: Choose an item.

Declaration of Competing Interests

Please complete a declaration of competing interests, considering the following questions:

- Have you in the past five years received reimbursements, fees, funding, or salary from an organisation that may in any way gain or lose financially from the publication of this manuscript, either now or in the future?
- Do you hold any stocks or shares in an organisation that may in any way gain or lose financially from the publication of this manuscript, either now or in the future?
- Do you hold or are you currently applying for any patents relating to the content of the manuscript?
- Have you received reimbursements, fees, funding, or salary from an organization that holds or has applied for patents relating to the content of the manuscript?
- Do you have any other financial competing interests?
- Do you have any non-financial competing interests in relation to this paper?

If you can answer no to all of the above, write 'I declare that I have no competing interests' below. If your reply is yes to any, please give details below.

I declare that I have no competing interests

I agree to the open peer review policy of the journal. I understand that my name will be included on my report to the authors and, if the manuscript is accepted for publication, my named report including any attachments I upload will be posted on the website along with the authors' responses. I agree for my report to be made available under an Open Access Creative Commons CC-BY license

(http://creativecommons.org/licenses/by/4.0/). I understand that any comments which I do not wish to be included in my named report can be included as confidential comments to the editors, which will not be published.

Choose an item.

To further support our reviewers, we have joined with Publons, where you can gain additional credit to further highlight your hard work (see: https://publons.com/journal/530/gigascience). On publication of this paper, your review will be automatically added to Publons, you can then choose whether or not to claim your Publons credit. I understand this statement.

Yes Choose an item.
