# Supplementary material for: IDseq—An open source cloud-based pipeline and analysis service for metagenomic pathogen detection and monitoring
Source: Gigascience. 2020 Oct 15;9(10):giaa111. doi: 10.1093/gigascience/giaa111 (PMC7566497; doi:10.1093/gigascience/giaa111)
Supplement: giaa111_Reviewer_3_Report_Original_Submission — Neda Tavakoli -- 5/31/2020 Reviewed [file giaa111_reviewer_3_report_original_submission.pdf]

Reviewer Report

Title: IDseq â€“ An Open Source Cloud-based Pipeline and Analysis Service for Metagenomic Pathogen Detection and Monitoring

Version: Original Submission **Date:** 5/31/2020

Reviewer name: Neda Tavakoli

Reviewer Comments to Author:

Summary:
The goal of this study is to provide an open source cloud-based metagenomics pipeline and service for global pathogen detection and monitoring from raw next-generation sequencing (NGS) reads. Their platform is optimized for scalable Amazon Web Services (AWS) cloud deployment. The authors provided a portal, IDseq portal to get raw mNGS data as input and to generate the assignment of reads and contigs to taxonomic categories.
This paper mainly presents two key contributions:
1. Providing an open source portal for pathogen detection from raw next-generation sequencing (NGS).
2. Providing open source Github repos.

Comments:
The paper makes a good effort to introduce an open source platform for pathogen detection. However, the provided Github is not working on a cloud platform other than AWS. I chose two platform to test the Github code on powerful cloud severs, however, the coded seems not working. These are the samples of errors:

```
ERROR: test_many_samples (tests.test_samples_on_local_steps.TestSamplesOnLocalSteps)
tests/test_utils.py", line 84, in run_step_and_match_outputs
  test_bundle, output_dir_s3)
idseq-dag/tests/idseq_step_setup.py", line 82, in get_test_step_object
  command.make_dirs(result_dir_local)
mkdir(name, mode)
PermissionError: [Errno 13] Permission denied: '/mnt/idseq'
```

We highly suggest the authors to fix these issue, and make their Github repo easy to work and install.

Level of Interest

Please indicate how interesting you found the manuscript: Choose an item.

Quality of Written English

Please indicate the quality of language in the manuscript: Choose an item.

Declaration of Competing Interests

Please complete a declaration of competing interests, considering the following questions:

- Have you in the past five years received reimbursements, fees, funding, or salary from an organisation that may in any way gain or lose financially from the publication of this manuscript, either now or in the future?
- Do you hold any stocks or shares in an organisation that may in any way gain or lose financially from the publication of this manuscript, either now or in the future?
- Do you hold or are you currently applying for any patents relating to the content of the manuscript?
- Have you received reimbursements, fees, funding, or salary from an organization that holds or has applied for patents relating to the content of the manuscript?
- Do you have any other financial competing interests?
- Do you have any non-financial competing interests in relation to this paper?

If you can answer no to all of the above, write 'I declare that I have no competing interests' below. If your reply is yes to any, please give details below.

I declare that I have no competing interests

I agree to the open peer review policy of the journal. I understand that my name will be included on my report to the authors and, if the manuscript is accepted for publication, my named report including any attachments I upload will be posted on the website along with the authors' responses. I agree for my report to be made available under an Open Access Creative Commons CC-BY license (http://creativecommons.org/licenses/by/4.0/). I understand that any comments which I do not wish to be included in my named report can be included as confidential comments to the editors, which will not be published.

Choose an item.

To further support our reviewers, we have joined with Publons, where you can gain additional credit to further highlight your hard work (see: https://publons.com/journal/530/gigascience). On publication of this paper, your review will be automatically added to Publons, you can then choose whether or not to claim your Publons credit. I understand this statement.

Yes Choose an item.
